# Use of ambr^®^250 to assess mucic acid production in fed-batch cultures of a marine *Trichoderma* sp. D-221704

**DOI:** 10.1186/s13568-022-01436-4

**Published:** 2022-07-13

**Authors:** Anu Tamminen, Rosaliina Turunen, Dorothee Barth, Virve Vidgren, Marilyn G. Wiebe

**Affiliations:** VTT Technical Research Centre of Finland Ltd, Tietotie 2, P.O. Box 1000, 02044 Espoo, Finland

**Keywords:** Mucic acid, Marine fungi, *Trichoderma*, Galactaric acid, D-galacturonic acid, Ambr®250

## Abstract

Mucic acid, a diacid with potential use in the food, cosmetic, chemical and pharmaceutical industries, can be produced by microbial conversion of D-galacturonic acid, which is abundant in pectin. Using the ambr^®^250 bioreactor system, we found that a recently generated transformant (D-221704, formerly referred to as T2) of a marine *Trichoderma* species produced up to 53 g L^−1^ mucic acid in glucose-limited fed-batch culture with D-galacturonic acid in the feed at pH 4, with a yield of 0.99 g mucic acid per g D-galacturonic acid consumed. Yeast extract was not essential for high production, but increased the initial production rate. Reducing the amount of glucose as the co-substrate reduced the amount of mucic acid produced to 31 g L^−1^. Mucic acid could also be produced at pH values less than 4.0 (3.5 and 3.0), but the amount produced was less than at pH 4.0. Furthermore, the yield of mucic acid on D-galacturonic acid at the end of the cultivations (0.5 to 0.7 g g^−1^) at these low pH levels suggested that recovery may be more difficult at lower pH on account of the high level of crystal formation. Another strain engineered to produce mucic acid, *Trichoderma reesei* D-161646, produced only 31 g L^−1^ mucic acid under the conditions used with D-221704.

## Key points

- Trichoderma D-221704 produced 53 g L^−1^ mucic acid in fed batch culture at pH 4.0
- Yeast extract was beneficial, but not essential for good mucic acid production
- The ambr^®^250 gave good, reproducible fed-batch cultivations with *Trichoderma *sp.

## Introduction

Fungi are efficient biocatalysts, converting simple substrates into more desirable products such as citric, gluconic and other organic acids, polyols, antibiotics, steroids, and other biologically active compounds. Many of these processes start from glucose, but D-galacturonic acid is also an interesting compound for bioconversion. It is available in relatively large quantities in the pectin found in citrus peel and sugar beet pulp, only part of which is needed or can be used in food products. Several publications have reported the use of genetically engineered fungi to convert D-galacturonic acid to mucic (galactaric) acid (Mojzita et al. 2010; Kuivanen et al. 2016, 2019; Barth and Wiebe 2017; Vidgren et al. 2020b). Mucic acid is a symmetrical hexaric acid i.e. a sugar acid with two terminal carboxyl groups. It is used as a chelator in some skincare products and can be reduced to adipic acid (Li et al. 2014), a precursor for Nylon, or converted to 2,5-furandicarboxylic acid (FDCA, Thomas et al. 2015). FDCA has the potential to replace the oil-based terephthalic acid in plastic bottles for carbonated soft drinks and a variety of other plastics (De Jong et al. 2012).

When developing strains for production of mucic acid, screening is typically done in small scale microtiter plates or flasks (Zhang et al. 2016; Paasikallio et al. 2017; Kuivanen et al. 2019). These are suitable to demonstrate production up to about 12 g L^−1^ mucic acid (Zhang et al. 2016; Paasikallio et al. 2017; Kuivanen et al. 2019). Production in small scale batch cultures may be limited because of sequential use of the co-substrate and the D-galacturonic acid (Mojzita et al. 2010) or by oxygen limitation. In fed-batch cultures, concentrations of 20 (Barth and Wiebe 2017; Paasikallio et al. 2017) to 25 (Vidgren et al. 2020b) g L^−1^ have been obtained with the filamentous fungi *Trichoderma reesei* D-161646 and a marine *Trichoderma* sp. LF328 T2 (VTTCC D-221704). The bioconversion process with D-161646 was successfully scaled up to 250 L, but the authors acknowledged that 20 g L^−1^ was still a low concentration for an efficient process, even though mucic acid can readily be precipitated from the solution at low pH (Paasikallio et al. 2017). The recently developed T2 strain may have higher production potential than D-161646 (Vidgren et al. 2020b), but conditions for good production were not explored.

Small scale fed-batch cultivations are possible in microtiter plates using the BioLector system, which allows feeding in cultures of 0.8 to 1.5 mL, but to obtain sufficient samples for analysis a larger system is needed. Bareither et al. (2013) described a small scale stirred bioreactor system, which has since become known as the ambr^®^250, and which was suitable for high cell density *Pichia pastoris* and *Escherichia coli* cultures, as well as mammalian cell (CHO) cultures. The system was well suited to controlled fed-batch cultivation and provided results comparable to 3 and 30 L cultures with the same organisms (Bareither et al. 2013). Ambr^®^250 reactors have since been used to demonstrate systematic experimental workflows for optimizing recombinant protein production in *Escherichia coli* (Tai et al. 2015), to scale down CHO cultivations (Manahan et al. 2019; Zhang et al. 2019), to develop control strategies (Hoshan et al. 2019) and elucidate effects of pH and temperature on antibody production in CHO systems (Wilson et al. 2019). Since ambr^®^250 has been used for various high cell density cultures, we considered whether it could be suitable for assessing production of mucic acid by a filamentous fungus. Fungal mucic acid cultivations are not high cell density, but the fungal mycelium causes the culture broth to be more viscous than bacterial or yeast fermentations. It can also make sampling difficult, but wide bore tips are available for sampling viscous suspensions in the ambr^®^250.

We describe here an assessment of pH, yeast extract and co-substrate concentrations in fed-batch cultivation to produce mucic acid from D-galacturonic acid using the marine *Trichoderma* sp. LF328 T2 (D-221704) that was genetically engineered to produce mucic acid by incorporating a uronate dehydrogenase gene under a strong synthetic promoter that would not be subject to glucose repression (Vidgren et al. 2020b). The cultivations were carried out in 110–150 mL cultures using an ambr^®^250 system, demonstrating its suitability for use with a filamentous fungus.

## Materials and methods

### Strains

Strain D-221704 (VTT culture collection, formerly referred to as T2) was engineered from *Trichoderma sp* LF328 (courtesy of Prof. Dr. Johannes Imhoff, GEOMAR Helmholtz Centre for Ocean Research Kiel, Germany) by inserting a gene encoding uronate dehydrogenase under a strong synthetic promoter in the D-galacturonate reductase gene (*gar2*), as described by Vidgren et al. (2020b). Spore suspensions were prepared by cultivating the fungus on potato-dextrose agar (BD, Sparks, Maryland, USA) for 7 days, after which the spores were collected from the surface of the culture in a solution containing 0.8% (w/v) NaCl, 0.025% (v/v) Tween20 and 20% (v/v) glycerol and stored at −80 °C. *Trichoderma reesei* QM6a Δ*gar 1 udh* (VTT D-161646, VTT culture collection) was obtained from the VTT culture collection for comparison.

### Media

The low phosphate medium described by Barth and Wiebe (2017) was used, with concentrations of D-glucose, D-galacturonate and yeast extract adjusted as needed. Phosphate elutes from the Aminex HPX-87H organic acid analysis column soon after D-galacturonate, resulting in overlapping peaks, so the analysis of D-galacturonate is more accurate when phosphate concentration is low. The medium for pre-cultures contained 20 g L^−1^ glucose as carbon source, with 2 g L^−1^ yeast extract and 4 g L^−1^ agar. The basic medium for the batch phase in bioreactors contained 20.8 ± 0.1 g L^−1^ glucose as carbon source, 4.6 ± 0.0 g L^−1^ D-galacturonic acid as substrate for mucic acid production and 2 g L^−1^ yeast extract. The feed for bioreactors contained 32 ± 2 g L^−1^ glucose, 94 ± 1 g L^−1^ D-galacturonate, and 2 g L^−1^ yeast extract, except for cultures in which the concentration of glucose or yeast extract were assessed. Glucose was used as a co-substrate to produce mucic acid with D-221704. To assess pH and the concentration of yeast extract, glucose was supplied at 32 ± 2 g L^−1^ in the feed (34% of the D-galacturonic acid concentration). Yeast extract was omitted from or provided at 0.5 g L^−1^ and 1.0 g L^−1^ in both batch and feed medium when assessing its effect on mucic acid production. In order to assess whether the amount of glucose was sufficient or excessive, mucic acid production was also tested in cultures in which the glucose concentration was reduced to 10.6 ± 0.5 g L^−1^ (11% of the D-galacturonic acid concentration) or increased to 50 ± 2 g L^−1^ (53% of the D-galacturonic acid concentration). The concentration of yeast extract in the batch medium and feed of these cultures with altered glucose concentration was reduced to 1 g L^−1^.

### Culture conditions

Pre-cultures for bioreactor inocula were grown in Erlenmeyer flasks (250 ml containing 50 mL medium) inoculated with approximately 10^6^ spores mL^−1^, final concentration. Flasks were incubated at 28 °C with 200 rpm agitation for 3 days before being used as inoculum for the bioreactors. Ambr^®^250 cultivations were inoculated with 20 mL of mycelial suspension per 90 mL medium.

Bioreactor cultures were grown in ambr^®^250 vessels, with an initial volume of 110 mL. Cultures were maintained at 35 °C (Barth and Wiebe 2017) with 1100 rpm and air flow of 100 mL min ^−1^. The pH was maintained at pH 3.0, pH 3.5 or pH 4.0, as indicated in the results, using 0.5 N NaOH and 0.5 N H_2_SO_4_. CO_2_ and O_2_ concentrations were continuously analyzed during the fermentation. Feed was provided at a constant rate of 0.35 mL h^−1^ during the feeding phase, which started approximately 13 h after inoculation, when the dissolved oxygen tension was below 30%. Cultures were fed for 240–310 h, after which the feed was stopped to allow utilization of any residual D-galacturonic acid. Samples (5 mL) were taken daily and maintained at +4 °C in the ambr^®^250 chiller until they could be processed further. Samples were processed as soon as possible after sampling of all vessels was complete. Samples were warmed to room temperature before further handling to allow mucic acid crystals which had precipitated at 4 °C to re-dissolve.

Each condition tested in our experiments was assessed in duplicate: all conditions were assessed in each of two independent ambr^®^250 cultivations. Online data from the cultivations are available at Zenodo (Tamminen et al. 2022). The concentration of D-galacturonate in the feed of the second set of cultivations was lower than in the first set, resulting in lower concentrations of mucic acid (Tamminen et al. 2022) in some conditions of the second set compared to the first.

### Analytics

The concentrations of D-galacturonic acid, glucose and mucic acid were assessed by HPLC using a Fast Acid Analysis Column (100 × 7.8 mm, BioRad Laboratories, Hercules, CA) linked to an Aminex HPX-87H organic acid analysis column (300 × 7.8 mm, BioRad laboratories) with 5 mM H_2_SO_4_ as eluent and a flow rate of 0.5 mL min^−1^. The column was maintained at 55 °C. Peaks were detected using a Waters 410 differential refractometer and a Waters 2487 dual wavelength UV (210 nm) detector. Samples were diluted with eluent to give expected concentrations of mucic acid between 1 and 4 g L^−1^ and heated at 105 °C for 1 h to solubilize crystals of galactaric acid before HLPC analysis (Paasikallio et al. 2017). The concentrations of mucic acid and yields reported here have been adjusted to take into account evaporation during the cultivations, based on the final volume of the reactor compared to the expected volume determined from the amount of feed and alkali added and the samples removed.

Biomass was analyzed in duplicate by centrifuging 1 mL samples at room temperature and washing twice with an equal volume of distilled water to solubilize and remove any residual crystals of mucic acid which had sedimented with the biomass. Washed biomass was dried at 105 °C overnight.

Data are presented as mean ± standard error of the mean. Analysis of Variance was used to compare production in three or more conditions, with significant differences identified using Fisher's multiple range test. The student-t test was used for comparisons of two strains or conditions.

Data are available at Zenodo (Tamminen et al. 2022).

## Results

### ***Production of mucic acid in Ambr***^***®***^***250 bioreactors at pH 4.0***

**Fig. 1:**
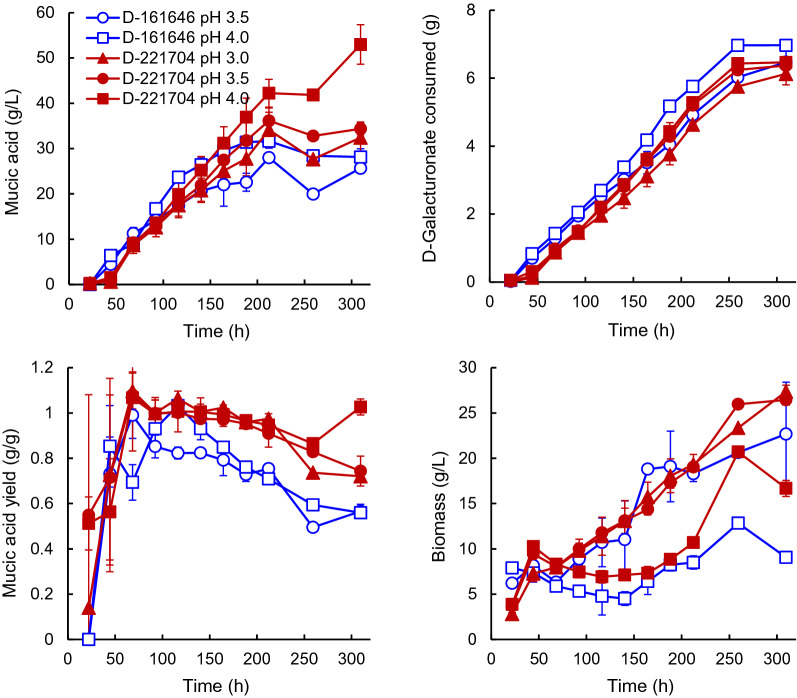
Concentrations of mucic acid and biomass produced and total amount of galacturonic acid consumed, along with the yield of mucic acid on D-galacturonic acid for cultures of D-221704 (solid symbols) and D-161646 (open symbols) grown in fed-batch culture at pH 4.0 (square), 3.5 (circle) or 3.0 (triangle). Error bars represent ± standard error of the mean for 2 cultures

The control strain, *T. reesei* D-161646 produced 31.4 ± 1.2 g L^−1^ mucic acid in 188 h when grown in fed-batch culture at pH 4.0 with 94 ± 2 g L^−1^ D-galacturonate and 32 ± 1 g L^−1^ glucose in the feed (Fig. 1). No further production occurred after 188 h (Fig. 1), although D-galacturonate continued to be consumed. The yield of mucic acid on D-galacturonic acid was highest (1.03 g g^−1^) around 116 h when there was 5 ± 0.5 g L^−1^ residual D-galacturonate in the supernatant. The yield of mucic acid on D-galacturonic acid decreased to 0.6 g g^−1^ after production stopped (Fig. 1).

Marine *Trichoderma* sp. D-221704 produced 36.9 ± 4.2 g L^−1^ mucic acid in 188 h and continued to produce for a further 120 h, to a final concentration of 53.0 ± 4.4 g L^−1^ at 309 h after inoculation (Fig. 1). As with D-161646, there was residual D-galacturonate (3–5 g L^−1^) in the broth during the first ~ 150 h feeding, but no residual D-galacturonate at the end of the cultivation. The yield of mucic acid on D-galacturonate was 0.99 ± 0.02 g g^−1^ throughout most of the feeding phase (68–309 h). Both strains initially (up to 140 or 160 h) produced mucic acid at a rate of 0.24 g L^−1^ h^−1^.

Biomass production and D-galacturonate consumption were similar for D-161646 and D-221704 (Fig. 1), although D-161646 (0.36 ± 0.01 h^−1^) had a higher specific growth rate than D-221704 (0.21 ± 0.01 h^−1^; Table 1), based on CO_2_ production.

**Table 1:** Specific growth rates (h^−1^) measured from CO_2_ output during the batch growth phase for D-221704 and D-161646 in the ambr^®^250 system

	Specific growth rate (h^−1^)	
	*Trichoderma* sp. D-221704	*T. reesei* D-161646
pH 4	0.21 ± 0.01^a^	0.36 ± 0.01^a^
pH 3.5	0.21 ± 0.02^a^	0.27 ± 0.002^b^
pH 3.0	0.15 ± 0.001^b^	
0 g L^−1^ YE	0.13 ± 0.001^a^	
0.5 g L^−1^ YE	0.18 ± 0.01^b^	
1 g L^−1^ YE	0.18 ± 0.01^b^	
2 g L^−1^ YE	0.21 ± 0.01^c^	

Values in the same column and category (either pH or yeast extract, YE, concentration) with the same superscript letter (a, b or c) did not differ significantly (p > 0.05)

### Production of mucic acid at pH 3.0 or 3.5

*T. reesei* D-161646 produced 28 ± 1 g L^−1^ mucic acid at pH 3.5 and D-221704 produced 36 ± 3 g L^−1^ (Fig. 1). As at pH 4, there was 3–5 g L^−1^ residual D-galacturonic acid in the culture broth during most of the feeding phase, but not during the final 80 h. The initial production rate was reduced to 0.17 ± 0.01 g L^−1^ h^−1^ for D-161646 and to 0.21 ± 0.03 g L^−1^ h^−1^ for D-221704, compared to the rates observed at pH 4.0. Both strains stopped producing mucic acid after 212 h, although D-galacturonic acid continued to be consumed (Fig. 1). The yield of mucic acid on D-galacturonic acid decreased for both strains after production stopped (Fig. 1, from 0.8 ± 0.03 to 0.6 ± 0.03 g g^−1^for D-161646 and from 0.97 ± 0.02 to 0.7 ± 0.07 g g^−1^ for D-221704).

**Fig. 2:**
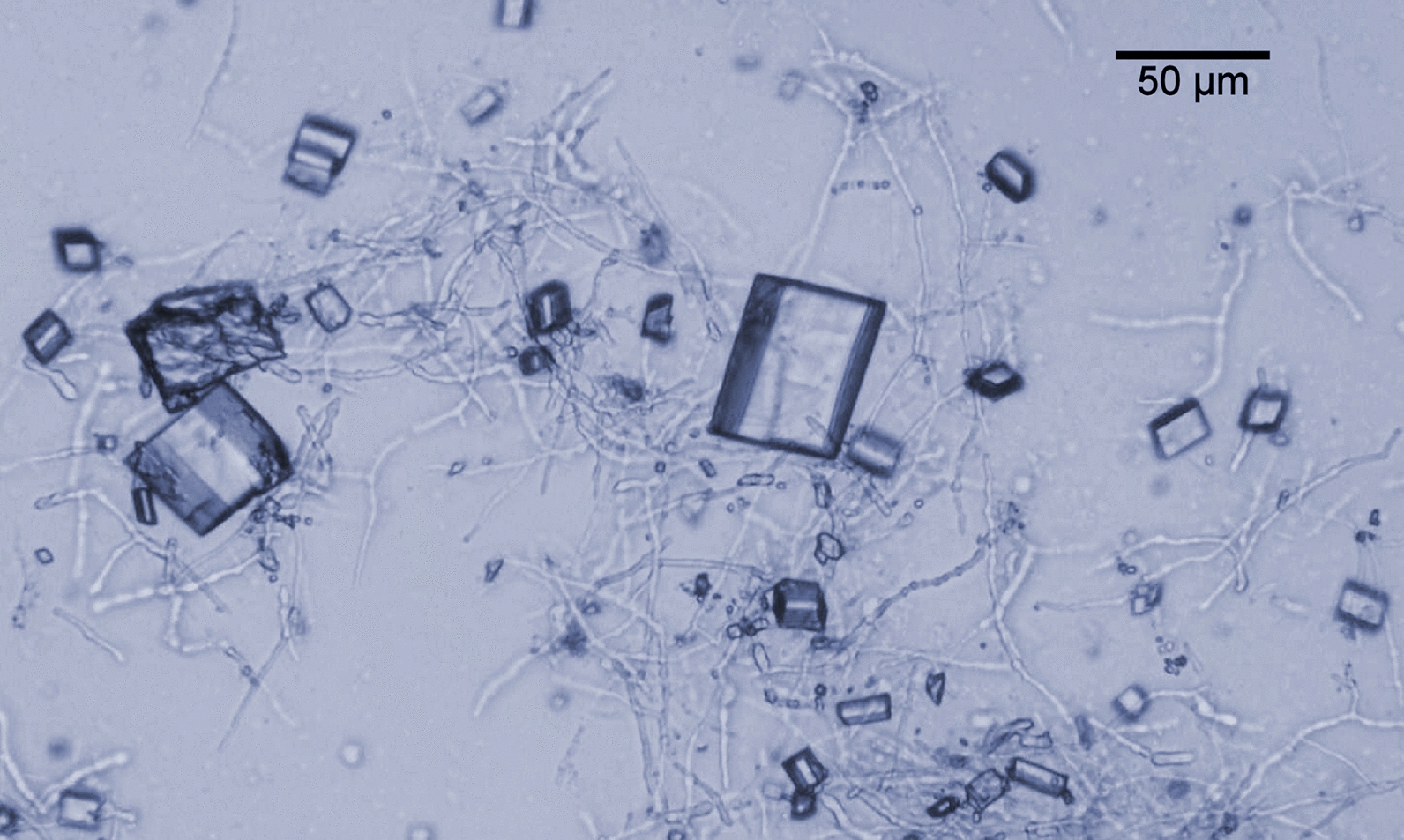
Mycelia of D-221704 growing at pH 3.5 in fed-batch culture, with crystals of mucic acid. Scale bar represents 50 µm

D-221704 produced 34 ± 4 g L^−1^ mucic acid at pH 3.0 at a rate of 0.19 ± 0.03 g L^−1^ h^−1^ and an average yield of 1.00 ± 0.02 g g^−1^ during the productive phase, decreasing to 0.7 ± 0.02 g^−1^ after production stopped (Fig. 1). Crystals of mucic acid were readily observed at pH 3.0 and 3.5 (Fig. 2), and it is possible that not all mucic acid was solubilized during the heating phase of sample handling.

Both strains produced more biomass at pH 3.5 (and at pH 3.0 for D-221704) than at pH 4.0. The specific growth rate of D-161646 was reduced to 0.27 ± 0.002 h^−1^ at pH 3.5, compared to pH 4, whereas D-221704 still grew at 0.21 ± 0.02 h^−1^ at pH 3.5, with a reduction to 0.14 ± 0.01 h^−1^ at pH 3.0 (Table 1).

### Effect of yeast extract on production of mucic acid with D-221704

**Fig. 3:**
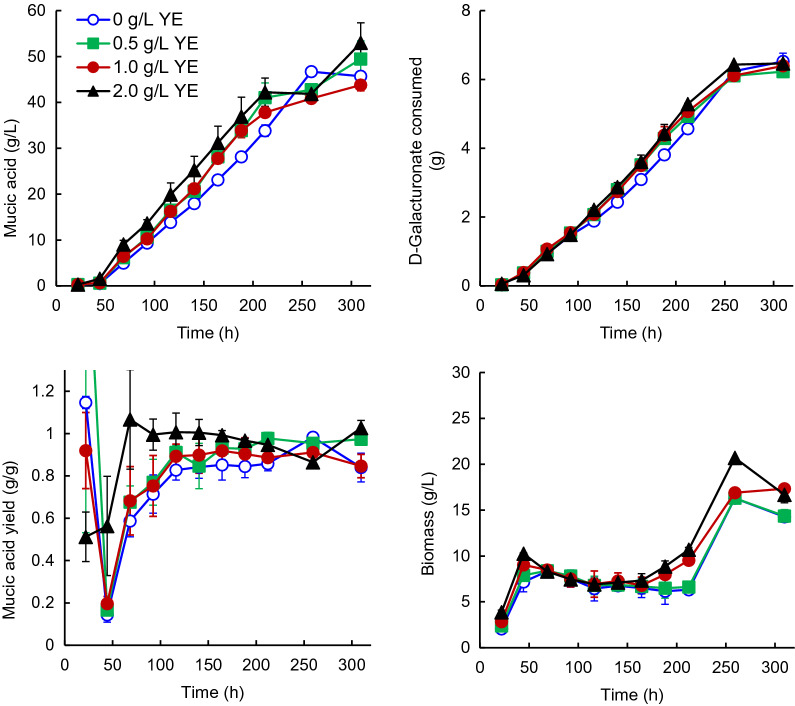
Concentrations of mucic acid and biomass produced and total amount of galacturonic acid consumed, along with the yield of mucic acid on D-galacturonic acid for cultures of D-221704 grown in fed-batch culture at pH 4.0 with 0 (open circle), 0.5 (solid square), 1.0 (solid circle) or 2.0 (solid triangle) g L^−1^ yeast extract. Error bars represent ± standard error of the mean for 2 cultures

Based on media developed for D-161646 (Barth and Wiebe 2017), the standard medium used to produce mucic acid with D-221704 contained 2 g L^−1^ yeast extract in both the batch phase and in the feed. Reducing the concentration of yeast extract in the medium, reduced the amount of mucic acid produced (43.8 ± 1.2, 49.5 ± 3.9, and 46.0 ± 0.7 g L^−1^ mucic acid with 1, 0.5 or 0 g L^−1^ yeast extract, respectively; Fig. 3), compared to that produced with 2 g L^−1^ yeast extract (53.0 ± 4.4 g L^−1^), although the reduction was not significant (p > 0.05) because of the variation in production observed with 2 g L^−1^ yeast extract. The initial production rate (0.23 ± 0.01 g L^−1^ h^−1^) was not affected by reducing the yeast extract to 0.5 g L^−1^ (p > 0.05), and was only slightly lower (0.20 ± 0.002 g L^−1^ h^−1^, p < 0.05 t-test) when no yeast extract was added. The yield of mucic acid on galacturonic acid was slightly reduced (p < 0.05, ANOVA) by reducing the concentrations of yeast extract from 2 (0.99 g g^−1^) to 0.5–1 g L^−1^ (0.90–0.92 g g^−1^) or to 0 g L^−1^ (0.84 g g^−1^; Fig. 3). Biomass production and galacturonate consumption were similar with all concentrations of yeast extract, although the specific growth rate was reduced (p < 0.05, ANOVA) as the amount of yeast extract was reduced (Table 1).

### Effect of co-substrate concentration on mucic acid production with D-221704

**Fig. 4:**
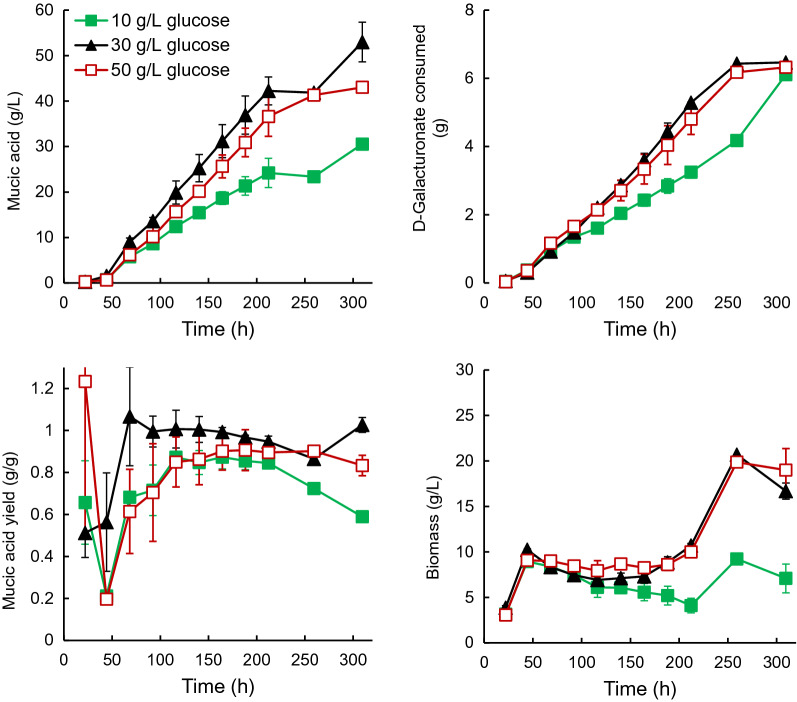
Concentrations of mucic acid and biomass produced and total amount of galacturonic acid consumed, along with the yield of mucic acid on D-galacturonic acid for cultures of D-221704 grown in fed-batch culture at pH 4.0 with 10 (solid square), 30 (solid triangle), or 50 (open square) g L^−1^ glucose in the feed, which also contained 94 g L^−1^ D-galacturonic acid and 1 g L^−1^ yeast extract. Error bars represent ± standard error of the mean for 2 cultures

Increasing the glucose concentration in the feed resulted in a reduction (not significant p > 0.05) in mucic acid production (43.0 ± 0.0 g L^−1^) and in the production rate (0.21 ± 0.03 g L^−1^ h^−1^), without affecting D-galacturonic acid uptake or biomass concentration (Fig. 4). In contrast, reducing the glucose concentration resulted in significantly (p < 0.05, ANOVA) less mucic acid production (30.6 ± 1.2 g L^−1^), at only 0.15 ± 0.02 g L^−1^ h^−1^. D-Galacturonic acid consumption was also reduced and the biomass concentration in the reactor declined during the feeding phase (Fig. 4). The yield of mucic acid on D-galacturonate was reduced (p < 0.05) when the glucose concentration was either reduced (0.84 ± 0.02 g g^−1^) or increased (0.85 ± 0.05 g g^−1^), compared to the provision as 34% of the D-galacturonate concentration.

## Discussion

Using the ambr^®^250 robotic bioreactor system, *Trichoderma* sp. LF328 D-221704 demonstrated its potential to produce 53 g L^−1^ mucic acid from D-galacturonic acid at a yield of 0.99 g g^−1^, which is close to the theoretical yield of 1.08 g g^−1^. This confirmed that the strain was more productive than the *T. reesei* strain D-161646 (Barth and Wiebe 2017), as suggested by Vidgren et al. (2020b).

The concentration of D-galacturonic acid in the feed can limit the maximum concentration of mucic acid produced in a fed batch culture, since feeding increases the volume of the culture, diluting the product. Thus to assess whether D-221704 could produce high concentrations of mucic acid it was necessary to increase the concentration of D-galacturonic acid in the feed from 45–67 g L^−1^ (Vidgren et al. 2020b) to 88–103 g L^−1^ D-galacturonic acid. Mucic acid production by D-161646 (Barth and Wiebe 2017) in earlier reports may have been limited by the D-galacturonic acid feed, even though residual D-galacturonic acid was detected in the supernatant, so it was grown in similar conditions to D-221704 at pH 4.0 and at pH 3.5. Not all D-galacturonic acid was consumed, but both D-161646 (0.24 g L^−1^ h^−1^) and D-221704 (0.26 g L^−1^ h^−1^) consumed D-galacturonic acid at higher rates than previously observed (0.16 g L^−1^ h^−1^, Barth and Wiebe 2017 and 0.20 g L^−1^ h^−1^, Vidgren et al. 2020a, for D-161646 and D-221704 respectively). They both also produced more mucic acid than previously observed (53 compared to 25 g L^−1^ for D-221704 and 31 compared to 20 g L^−1^ for D-161646; Barth and Wiebe 2017; Vidgren et al. 2020b). Although several D-galacturonate transporters have been identified in fungi (Martens-Uzunova and Schaap 2008; Zhang et al. 2011; Benz et al. 2014; Sloothaak et al. 2014; Protzko et al. 2018), their transport mechanisms have not been well characterized. Benz et al. (2014) reported that the GAT-1 transporter of *Neurospora crassa* functions as a high affinity transporter in *Saccharomyces cerevisiae*, but low affinity transporters have not been identified. In *S. cerevisiae* low affinity uptake of D-galacturonate at low pH has been observed, but the transporter(s) was not identified (Souffriau et al. 2012). That D-galacturonic acid was utilized more rapidly by both D-221704 and D-161646 when supplied in excess may suggest that low affinity transport was important. Thus D-galacturonic acid availability may still have limited the production of mucic acid in these strains and further improvements in both rate and titer may be possible with higher concentrations of D-galacturonic acid in the feed. This would require pre-hydrolysis if pectin were used as the feed, since pectin solutions become viscous and may form gels at high concentration.

Although D-161646 produced more mucic acid (31 g L^−1^) than previously reported, D-221704 produced even more (53 g L^−1^). D-221704 and D-161646 produced mucic acid at similar rates, but D-221704 continued to produce mucic acid for longer than D-161646. This may indicate D-221704 has more tolerance to the acid. *Trichoderma reesei* did not grow well in the presence of mucic acid at low pH (Barth and Wiebe 2017). However, the continued removal of D-galacturonate from the culture broth, without apparent production of mucic acid, may indicate that it was harder to solubilize mucic acid in the D-161646 cultures than in the D-221704 cultures and that thus not all mucic acid that was produced was measured. Barth and Wiebe (2017) observed that the composition of the medium affected the solubility of mucic acid in the culture broth. Although both strains were grown in the same medium, with the same feed, they differed in the amount of acid and alkali required to maintain the pH: D-161646 required more acid than D-221704, and less base at pH 4.0 (Tamminen et al. 2022). The same method of diluting and solubilizing mucic acid crystals in the HPLC eluent (Paasikallio et al. 2017) was applied to all samples, but the method has only previously been used for concentrations of mucic acid up to ~ 25 g L^−1^. Failure to solubilize all mucic acid in some broths would explain the decrease in yield of mucic acid on D-galacturonic acid that was observed at the end of D-161646 cultures and cultures at low pH. There is no other evidence that either D-161646 or D-221704 have alternative pathways for metabolizing D-galacturonic acid, which would also account for the decrease in yield. Difficulty in separating mucic acid from mycelia could be resolved by Soxhlet extraction (Thomas et al. 2017), but this was impractical when handling the large number of small samples obtained from multiple parallel cultures in the ambr^®^250 system.

Barth and Wiebe (2017) found that mucic acid was produced better at pH 4.0 than at higher pH values, even though the solubility of mucic acid decreased at values less than 4.6. Production at pH values less than 4.0 was not assessed because of the low solubility of mucic acid. Here we demonstrated that mucic acid could also be produced at pH 3.5 or pH 3.0 (Fig. 1), although titer, yield and production rate were all reduced compared to production at pH 4.0. As with D-161646 compared to D-221704, more acid was added to sustain the pH of cultures at pH 3.0 or 3.5 than at pH 4.0 and this may have affected the solubilization of the mucic acid crystals. D-Galacturonic acid consumption and biomass production were good at pH 3.5 and pH 3.0. If mucic acid could be produced and recovered at these low pH values it would reduce the cost of acid addition to precipitate mucic acid during downstream processing.

Yeast extract also contributes considerably to the cost of producing mucic acid. It was added because Barth and Wiebe (2017) observed that its addition to *T. reesei* D-161646 cultures improved the production of mucic acid, probably by increasing its solubility and recovery. The observation was made in cultures using KOH for pH control and it was also noted that mucic acid was less soluble in the presence of excess potassium ions than with sodium or ammonium ions (Barth and Wiebe 2017). With D-221704 we found that the yeast extract concentration could be reduced to 0.5 g L^−1^ without affecting the titer or production rate (Fig. 3). Complete omission of yeast extract from the medium resulted in a 14% reduction in the mucic acid production rate and 12% reduction in titer, which would need to be balanced against the saving obtained by omitting the yeast extract.

Glucose was used here as co-substrate to produce biomass and provide energy for mucic acid production and/or export, since Vidgren et al. (2020b) had shown that glucose was a good co-substrate for D-221704. As observed by Paasikallio et al. (2017), commercial pectin sources may be contaminated with high concentrations of glucose. While mucic acid production would not use commercially available food-grade pectin, high glucose content could also result from hydrolysis of cellulose in sugar beet pulp or citrus peel, if pectin was not first extracted. A high glucose to D-galacturonic acid ratio had little impact on mucic acid production (Fig. 4). On the other hand, reducing the available co-substrate resulted in less biomass production (and therefore less biocatalyst), reduced mucic acid production and a lower production rate (Fig. 4). Thus there would be no advantage to using very pure pectin, since more co-substrate would then need to be added.

Both *Trichoderma* sp. D-221704 and *T. reesei* D-161646 grew well in the ambr^®^250 vessels. D-161646 had a specific growth rate (µ) of 0.36 h^−1^ at pH 4.0 and 35 °C. Although specific growth rates at 35 °C have not been reported for this strain, CO_2_ data from the 250 L pilot and 1 L cultures reported by Paasikallio et al. (2017) showed specific growth rates of 0.340 h^−1^ and 0.40 h^−1^ (unpublished data, available from Tamminen et al. 2022), respectively, confirming that growth in the ambr®250 vessel was comparable to that in larger vessels. The specific growth rate of D-221704 in the ambr®250 cultures (µ = 0.21 h^−1^) was lower than that of D-161646. Vidgren et al. (2020b) did not report the specific growth rate of T2 (= D-221704) in the 2 L bioreactors, but unpublished data (µ = 0.35 h^−1^ on glucose, Tamminen et al. 2022) demonstrates that it was expected to be similar to that observed for D-161646 at 35 °C. Biomass concentrations in the ambr^®^250 cultures were similar for the two strains when grown in the same condition (Fig. 1) and comparable to those published previously for T. reesei D-161646 (Paasikallio et al. 2017). Foam production was not a problem in these small scale cultures, but a low initial cultivation volume of only 110 mL was used in order to ensure a large head space in case foam production did occur and to avoid problems of splashing as the volume increased to the area of the upper impellor. The amount of evaporation was higher than expected and indicates that the settings for condensation at the lid should be further optimized. The wide bore sampling tips provided for the ambr^®^250 were adequate for sampling these cultures of filamentous fungi. However, further automation of sample handling for the biomass and HPLC analyses would be beneficial to increase the throughput made possible by having multiple parallel culture vessels available.

## Data Availability

The datasets supporting the conclusions of this article are included within the article and/or are available in the Zenodo repository (https://zenod.org/) (Tamminen et al. 2022).
